# Acetylcholinesterase Inhibitors: Pharmacology and Toxicology

**DOI:** 10.2174/1570159X11311030006

**Published:** 2013-05

**Authors:** Mirjana B Čolović, Danijela Z Krstić, Tamara D Lazarević-Pašti, Aleksandra M Bondžić, Vesna M Vasić

**Affiliations:** 1 Department of Physical Chemistry, Vinča Institute of Nuclear Sciences, University of Belgrade, Belgrade, Serbia; 2 University School of Medicine, Institute of Medical Chemistry, University of Belgrade, Belgrade, Serbia

**Keywords:** Acetylcholine, acetylcholinesterase, Alzheimer’s disease drugs, carbamates, detoxification, irreversible inhibitors, organophosphates, reversible inhibitors.

## Abstract

Acetylcholinesterase is involved in the termination of impulse transmission by rapid hydrolysis of the neurotransmitter acetylcholine in numerous cholinergic pathways in the central and peripheral nervous systems. The enzyme inactivation, induced by various inhibitors, leads to acetylcholine accumulation, hyperstimulation of nicotinic and muscarinic receptors, and disrupted neurotransmission. Hence, acetylcholinesterase inhibitors, interacting with the enzyme as their primary target, are applied as relevant drugs and toxins. This review presents an overview of toxicology and pharmacology of reversible and irreversible acetylcholinesterase inactivating compounds. In the case of reversible inhibitors being commonly applied in neurodegenerative disorders treatment, special attention is paid to currently approved drugs (donepezil, rivastigmine and galantamine) in the pharmacotherapy of Alzheimer’s disease, and toxic carbamates used as pesticides. Subsequently, mechanism of irreversible acetylcholinesterase inhibition induced by organophosphorus compounds (insecticides and nerve agents), and their specific and nonspecific toxic effects are described, as well as irreversible inhibitors having pharmacological implementation. In addition, the pharmacological treatment of intoxication caused by organophosphates is presented, with emphasis on oxime reactivators of the inhibited enzyme activity administering as causal drugs after the poisoning. Besides, organophosphorus and carbamate insecticides can be detoxified in mammals through enzymatic hydrolysis before they reach targets in the nervous system. Carboxylesterases most effectively decompose carbamates, whereas the most successful route of organophosphates detoxification is their degradation by corresponding phosphotriesterases.

## 1 CHOLINESTERASES

Cholinesterase is a family of enzymes that catalyzes the hydrolysis of the neurotransmitter acetylcholine (ACh) into choline and acetic acid, a reaction necessary to allow a cholinergic neuron to return to its resting state after activation. It involves two types:

1. Acetylcholinesterase (AChE, acetycholine acetylhydrolase, E.C. 3.1.1.7) is found in many types of conducting tissue: nerve and muscle, central and peripheral tissues, motor and sensory fibers, and cholinergic and noncholinergic fibers. The activity of AChE is higher in motor neurons than in sensory neurons [1-3]. AChE is also found in the red blood cell membranes, where it constitutes the Yt blood group antigen. The enzyme exists in multiple molecular forms, which possess similar catalytic properties, but differ in their oligomeric assembly and mode of attachment to the cell surface. In the mammalian brain the majority of AChE occurs as a tetrameric, G4 form (10) with much smaller amounts of a monomeric G1 (4S) form [4].
2. Pseudocholinesterase (BuChE, EC 3.1.1.8), also known as plasma cholinesterase, butyrylcholinesterase, or acylcholine acylhydrolase, is found primarily in the liver. Different from AChE, BuChE hydrolyzes butyrylcholine more quickly than ACh [5].

### 1.1 Acetylcholine as Neurotransmitter

The first neurotransmitter discovered ACh is neurotransmitter at all autonomic ganglia, at many autonomically innervated organs, at the neuromuscular junction, and at many synapses in the central nervous system. In the autonomic nervous system, ACh is the neurotransmitter in the preganglionic sympathetic and parasympathetic neurons, as well as at the adrenal medulla and serves as the neurotransmitter in all the parasympathetic innervated organs. ACh is also the neurotransmitter at the sweat glands, and at the piloerector muscle of the sympathetic autonomic nervous system. In the peripheral nervous system, ACh is the neurotransmitter at the neuromuscular junction between the motor nerve and skeletal muscle. In the central nervous system, ACh is found primarily in interneurons, and a few important long-axon cholinergic pathways have also been identified. Noteworthy is the cholinergic projection from the *nucleus basalis* of Meynert (in the basal forebrain) to the forebrain neocortex and associated limbic structures. Degeneration of this pathway is one of the pathologies associated with Alzheimer's disease (AD) [6, 7].

**Fig. (1):**
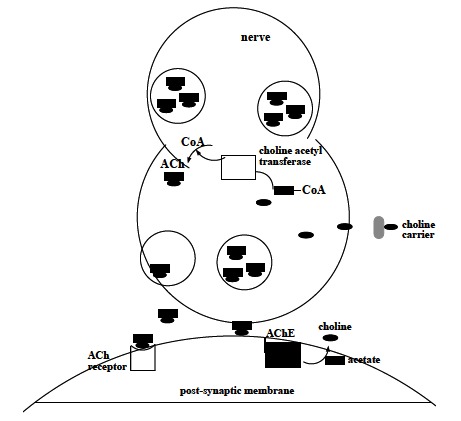
Mechanism of AChE action in neurotransmission.

ACh is synthesized by a single step reaction catalyzed by the biosynthetic enzyme choline acetyltransferase and the presence of this enzyme is the "marker" that a neuron is cholinergic. The majority of the ACh in nerve endings is contained in clear 100 nm vesicles and a small amount is also free in the cytosol. The uptake of ACh into storage vesicle occurs through an energy-dependent pump that acidifies the vesicle [7]. During neurotransmission, ACh is released from the nerve into the synaptic cleft and binds to ACh receptors (nicotinic and muscarinic) on the post-synaptic membrane, relaying the signal from the nerve. AChE, also located on the post-synaptic membrane, terminates the signal transmission by hydrolyzing ACh. The liberated choline from the ACh decomposition is taken up again by the pre-synaptic nerve and the neurotransmitter is synthesized by combining with acetyl-CoA through the action of choline acetyltransferase (Fig. **1**) [8, 9].

### 1.2 Acetylcholinesterase – Structure and Catalytical Function

AChE is a serine hydrolase mainly found at neuromuscular junctions and cholinergic brain synapses. Its principal biological role is termination of impulse transmission at cholinergic synapses by rapid hydrolysis of the neurotransmitter ACh to acetate and choline. AChE has a remarkably high specific catalytic activity, specially for a serine hydrolase - each molecule of AChE degrades about 25000 molecules of ACh per second, approaching the rate of a diffusion-controlled reaction [10, 11]. 

**Fig. (2):**
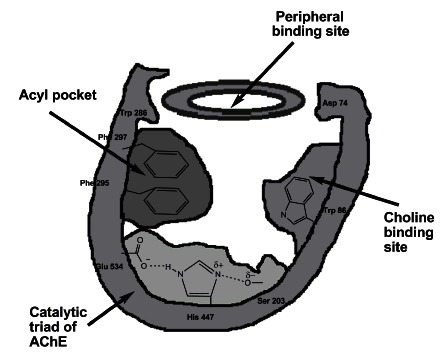
Schematic representation of AChE binding sites.

The molecule has an ellipsoidal shape with dimensions ~ 45 Ǻ by 60 Ǻ by 65 Ǻ. The enzyme monomer is an α/β protein containing 12 – stranded central mixed β sheet surrounded by 14 α helices. The most remarkable feature of the structure is a deep and narrow gorge, ~ 20 Ǻ long penetrating halfway into the enzyme and widens out close to its base [12]. The active site of AChE is located 4 Ǻ from the bottom of the molecule and comprises two subsites - the ″anionic″ subsite and ″esteratic″ subsite corresponding to the catalytic machinery and the choline-binding pocket, respectively [13]. The anionic subsite, uncharged and lipophilic, binds the positive quartenary amine of choline moiety of ACh, as well as both quartenary ligands (edrophonium, N-methylacridinium) acting as competitive inhibitors [14, 15], and quartenary oximes which effectively reactivate organophosphate-inhibited AChE [16]. The cationic substrates are not bound by a negatively-charged amino acid in the anionic site, but by interaction of 14 aromatic residues that line the gorge leading to the active site. [17-19]. All 14 amino acids in the aromatic gorge are highly conserved across different species [20]. Among the aromatic amino acids, tryptophan 84 is critical and its substitution with alanine causes a 3000-fold decrease in the enzyme activity [21]. The esteratic subsite, where ACh is hydrolyzed to acetate and choline, contains, similar to the catalytical subsites of other serine hydrolases, the catalytic triad of three amino acids: serine 200, histidine 440 and glutamate 327 (Fig. **2**). 

**Fig. (3):**
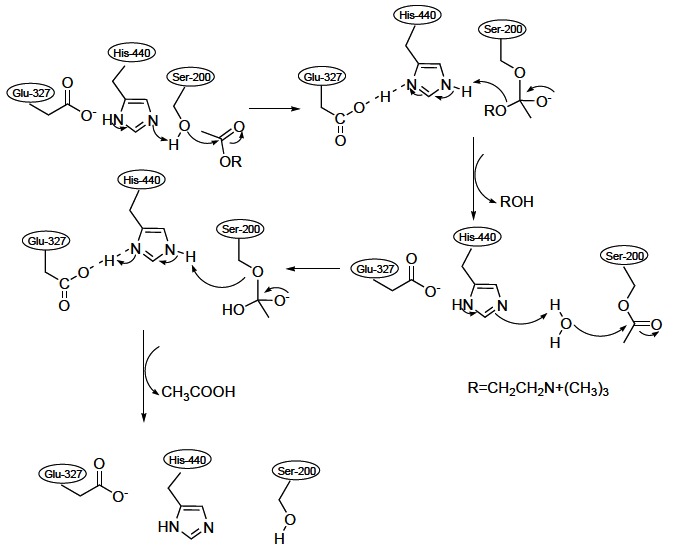
Mechanism of ACh hydrolysis catalyzed by AChE.

The hydrolysis reaction of the carboxyl ester leads to the formation of an acyl-enzyme and free choline. Then, the acyl-enzyme undergoes nucleophilic attack by a water molecule, assisted by the histidine 440 group, liberating acetic acid and regenerating the free enzyme (Fig. **3**) [13].

In addition to two subsites of active centre, AChE contains one or more ″peripheral″ anionic sites distinct from the choline-binding pocket of the active site (Fig. **2**). It serves for binding ACh and other quartenary ligands acting as uncompetitive inhibitors that bind at a site clearly distinct from that occupied by the monoquartenary competitive inhibitors [22]. This site is involved in the substrate inhibition characteristics of AChE [23]. 

Knowledge of AChE structure is essential for understanding its high catalytic efficacy and the molecular basis for the recognition of ACh by other ACh-binding protein (ACh receptors), as well as elucidation of the mechanism of action underlying the pharmacological and toxicological action of these agents for the purpose of rational drug design [24]. 

In this review pharmacological and toxicological relevant AChE inhibitors are summarized, mechanism of their action, and the ways of detoxification of irreversible and reversible AChE inactivators are discussed as well.

## 2 ACETYLCHOLINESTERASE INHIBITORS

AChE inhibitors or anti-cholinesterases inhibit the cholinesterase enzyme from breaking down ACh, increasing both the level and duration of the neurotransmitter action. According to the mode of action, AChE inhibitors can be divided into two groups: irreversible and reversible**. **Reversible inhibitors, competitive or noncompetitive, mostly have therapeutic applications, while toxic effects are associated with irreversible AChE activity modulators. 

### 2.1 Reversible Acetylcholinesterase Inhibitors

Reversible AChE inhibitors play an important role in pharmacological manipulation of the enzyme activity. These inhibitors include compounds with different functional groups (carbamate, quaternary or tertiary ammonium group), and have been applied in the diagnostic and/or treatment of various diseases such as: myasthenia gravis, AD, post-operative ileus, bladder distention, glaucoma, as well as antidote to anticholinergic overdose.

#### 2.1.1 Reversible Acetylcholinesterase Inhibitors in Alzheimer’s Disease Treatment

AD is a progressive neurological disorder, the most common form of dementia, characterized by memory loss and other intellectual abilities serious enough to interfere with daily life [25]. The disease is associated with loss of cholinergic neurons in the brain and the decreased level of ACh [26]. The major therapeutic target in the AD treatment strategies is the inhibition of brain AChE [26, 27]. There is no cure for AD, and reversible AChE inhibitors, employed in the therapy, treat symptoms related to memory, thinking, language, judgment and other thought processes. Actually, different physiological processes related to AD damage or destroy cells that produce and use ACh, thereby reducing the amount available to deliver messages to other cells. Cholinesterase inhibitor drugs, inhibiting AChE activity, maintain ACh level by decreasing its breakdown rate. Therefore, they boost cholinergic neurotransmission in forebrain regions and compensate for the loss of functioning brain cells. No drug has an indication for delaying or halting the progression of the disease [28]. Medications currently approved by regulatory agencies such as the U.S. Food and Drug Administration (FDA) and the European Medicines Agency (EMA) to treat the cognitive manifestations of AD and improve life quality of the patients are: donepezil, rivastigmine and galantamine as reversible AChE inhibitors, and memantine as an NMDA receptor antagonist [29, 30, 31]. Tacrine was the first of the AChE inhibitors approved for the AD treatment in 1993, but its use has been abandoned because of a high incidence of side effects including hepatotoxicity [32, 33]. 

##### Donepezil

**Fig. (4):**
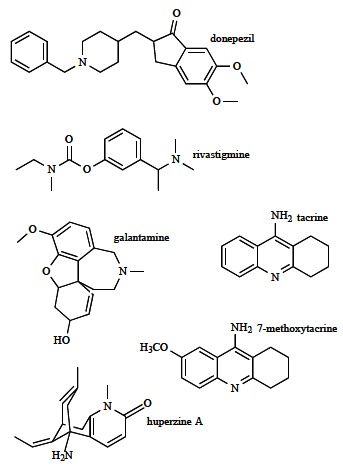
Selected reversible AChE inhibitors in pharmacotherapy of AD.

(Fig. **4**) is a selective, reversible AChE inhibitor that binds to the peripheral anionic site exerting not only symptomatic effects in the AD treatment, but also causative ones delaying the deposition of amyloid plaque [34, 35]. The drug is produced by pharmaceutical companies Eisai and Pfizer under the trade name Aricept. Although its principal therapeutic use is in the palliative treatment of mild to moderate AD, some clinical studies state that donepezil improves cognitive function in patients with severe AD symptoms as well [36]. It is available as disintegrating tablet and oral solution, being 100% oral bioavailability with ease crossing the blood-brain barrier and slow excretion. As it has a half-life of about 70 hours, it can be taken once a day. The drug is available in 5 and 10 mg dose strengths, and treatment is usually initiated at 5 mg per day, and increased after several weeks to 10 mg per day. Maximum daily dose is 23 mg once daily [37]. Patients receiving the higher dose showed mild improvement in cognitive functions, and no improvement on overall functioning. On the other hand, the higher drug dose induced the increased incidence of cholinergic side effects in patients, which limited its wider use [38]. Common donepezil adverse effects include gastrointestinal anomalies-nausea, diarrhea, anorexia, abdominal pain, as well as increase in cardiac vagal tone causing bradycardia [39]. Additionally, recent studies have suggested donepezil ability to improve speech in children with autism, while its indication in other cognitive disorders such as Lewy body dementia, schizophrenia and vascular dementia is not currently approved [40-42].

##### Rivastigmine

(Fig. **4**) (sold under the trade name Exelon) is a powerful, slow-reversible carbamate inhibitor that blocks cholinesterase activity through binding at the esteratic part of the active site. Unlike donepezil that selectively inhibits AChE, rivastigmine inhibits both BuChE and AChE. It has received approval for the treatment of mild-to-moderate AD in 60 countries including all member states of the European Union and the USA [43]. The drug is administered orally as capsules or liquid formulations, with good absorption and bioavailability of about 40% in the 3 mg dose. It is eliminated through the urine and has relatively few drug-drug interactions. The treatment is initiated at 1.5 mg twice daily, and is increased gradually over weeks to 6 mg twice daily; the increment is 3 mg per day every 2 to 4 weeks. Early and continued treatment of AD with rivastigmine maximizes the observed beneficial effects in the rate of decline of cognitive function, activities of daily living, and severity of dementia with daily doses of 6 to 12 mg. Adverse events are consistent with the cholinergic actions of the drug, and include nausea, vomiting, diarrhea, anorexia, headache, syncope, abdominal pain and dizziness [32, 44]. The side effects can be reduced using transdermal patch delivering rivastigmine. The target dose of 9.5 mg/day delivered by patch provides similar clinical effects (improved memory and thinking, activities of daily living, concentration) as the highest recommended doses of rivastigmine capsules, but with three times fewer reports of nausea and vomiting [45]. In addition to AD, rivastigmine can be applied in the treatment of Lewy bodies and Parkinson’s disease dementia [43, 46].

##### Galantamine

 (trade name Razadyne, Nivalin) is an alkaloid (Fig. **4**) isolated from the plant *Galanthus woronowii* being applied for the treatment of mild to moderate AD. It is a selective, competitive, rapidly-reversible AChE inhibitor that interacts with the anionic subsite, as well as with the aromatic gorge [47-49]. Besides, the drug is an allosteric ligand at nicotinic cholinergic receptors inducing their modulation. It interacts with the nicotinic receptor at binding sites separate from those for ACh and nicotinic agonists, and acts specifically to enhance the activity (sensitize) of nicotinic receptors in the presence of ACh [50, 51]. As the severity of cognitive impairment in AD correlates with loss of nicotinic receptors, this effect appears to be beneficial for the disorder treatment [52]. Galantamine absorption is rapid and complete, with absolute oral bioavailability between 80 and 100% and seven hours half-life. The treatment is usually initiated at 4 mg twice daily, and can be increased gradually up to 12 mg twice daily [39]. The drug side effects are similar to those of other AChE inhibitors, mainly with gastrointestinal symptoms. Galantamine seems to be less tolerated compared with the other AD drugs. However, a careful and gradual titration over more than three months may improve long-term tolerability [29]. Since galantamine has allosteric potentiating effects at nicotinic receptors, it affects not only cholinergic transmission but also other neurotransmitter systems such as monoamines, glutamate, and γ-aminobutyric acid (GABA) through its allosteric mechanism. These effects may result in more beneficial effects, and improve cognitive dysfunction and psychiatric illness in schizophrenia, major depression, bipolar disorder and alcohol abuse [53].

Considering the clinical effects of donepezil, rivastigmine and galantamine, there is no evidence that any of these medications is superior in terms of efficacy. However, donepezil has been found to be better tolerated, with less gastrointestinal side effects than rivastigmine or galantamine [39]. Beside the described drugs approved by FDA and EMA for the AD symptomatic treatment, new AChE inhibitors have been synthesized and tested. So, the derivative of hepatotoxic tacrine (Fig. **4**) - the potent inhibitor of AChE anionic active site, 7-methoxytacrine was widely studied as a suitable substitute to tacrine. *In vitro* and *in vivo* tests indicated both its less toxic effects and stronger inactivating power against AChE related to tacrine [54]. Furthermore, natural alkaloid huperzine A (Fig. **4**) is originated from the firmoss *Huperzia serrata*, and can be synthesized as well. The target of this AChE inhibitor is the peripheral anionic site, which makes the AD drug able to affect the symptoms as well as the cause of the disorder [55, 49] (see about donepezil above). The drug is a more potent AChE inhibitor than tacrine, galantamine and rivastigmine, while donepezil exhibits higher anti-AChE activity. Compared to other AChE inhibitors, huperzine A demonstrated better penetration through blood-brain barrier, higher oral bioavailability and longer AChE inhibition. Clinical trials with this AChE inhibitor revealed cognitive and functional impairments at patients with AD, schizophrenia and vascular dementia, and memory improvement of elder people [56]. In addition, protoberbrine alkaloids (berberine, palmatine, jatrorrhizine, epiberberine), as natural robust AChE inhibitors, are contemplated promising symptomatic therapeutic agents for AD [57]. It is necessary to emphasize that the pharmacological profile of the eutomer (bioactive enantiomer or enantiomer having higher pharmacological activity) and distomer (opposite to eutomer) of the chiral drugs (e.g. donepezil, rivastigmine, galantamine) is differential.

Looking for potent and selective AChE inhibitors as potential anti-Alzheimer drugs, new compounds have recently been designed, synthesized and tested. Novel donepezil-tacrine and oxoisoaporphine-tacrine congeners hybrid related derivatives, coumarin and huperzine A derivatives have exhibited high AChE inhibitory activity with IC_50_ values in the nanomolar range, and ability to bind simultaneously to both peripheral and catalytic sites of the enzyme. For the reason, these dual binding site inhibitors are promising compounds for developing disease-modifying drugs for the future treatment of AD [58-62], Additionally, new synthesized symmetrical bispyridinium and carbamate anti-AChE compounds inhibit the enzyme in micromolar concentrations, making them the potential candidates for the treatment of AD [63, 64]. 

Generally, the disadvantage of the AChE inhibitors in AD treatment is modest and temporary benefits lasting for a maximum 12-24 months. Actually, these drugs do not reduce the rate of decline in cognitive or functional capacities over the long term [65, 66]. Despite this fact, reversible AChE inhibitors provide meaningful symptomatic benefits, thereby remaining the mainstay of pharmacotherapy in AD. Moreover, their use is standard and supported by evidence [39].

#### 2.1.2 Carbamates 

Carbamates are organic compounds derived from carbamic acid (NH_2_COOH). The structure of biologically active carbamates is displayed in Fig. (**5**), where X can be oxygen or sulphur (thiocarbamate), R_1 _and R_2_ are usually organic or alkyl substituents, but R_1_ or R_2_ may also be hydrogen, and R_3 _is mostly an organic substituent or sometimes a metal. In addition to their use as therapeutic drugs in human medicine (AD, myasthenia gravis, glaucoma, Lewy bodies, Parkinson’s disease), these reversible AChE inhibitors have been applied as pesticides, then as parasiticides in veterinary medicine, and in prophylaxis of organophosphorus compounds (OPs) poisoning as well [67].

Since carbamates, as well as OPs, are AChE inhibitors, both compounds cause similar toxic acute effects and symptoms derived from poisoning. The principal difference between OP and carbamate induced inhibitory action is in the stability of the AChE-OP/carbamate complex. Actually, OPs are able to phosphorylate serine residues of AChE in non-reversible way (Fig. **6**), whereas the carbamylated serine residue is less stable and the carbamyl moiety can be split from the enzyme by spontaneous hydrolysis (decarbamylation time is 30-40 minutes) [68, 69]. Therefore, carbamates are considered reversible AChE inhibitors. Furthermore, carbamates, analogously to OPs, reversibly inhibit neuropathy target esterase, but, unlike OPs, are not able to dealkylate *i.e.* age the inhibited enzyme. So, carbamates are not delayed neuropathy inducers (see more about OP induced neuropathy below) [70, 71]. Moreover, they exhibit protective effects when applied before OPs induced neuropathy. Thereupon, the neuropathic OPs are not able to inhibit and age neuropathy target esterase previously reversibly inhibited by carbamates. On the other hand, carbamates stimulate delayed neuropathy or make it more severe, when they are dosed after applying OP neuropathic doses, inducing promotion of delayed neuropathy [72].

**Fig. (5):**
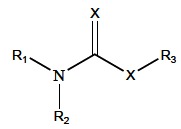
General chemical structure of biologically active carbamates.

Carbamate compounds are applied as fungicides, insecticides and herbicides in agriculture, and belong to the second group of pesticides inhibiting cholinesterases. Carbamates containing hydrogen and methyl group in the place of R_2 _and R_1_ (Fig. **5)**, respectively, exert the insecticide activity. Carbamate insecticides include aldicarb, carbofuran, carbaryl, fenobucarb, propoxur (Fig. **7**). Their insecticide killing action is based on reversible AChE inactivation. Carbamates are considered to be safer than OP insecticides that irreversibly inhibit AChE causing more severe cholinergic poisoning [67, 73-75]. It was found that stress conditions can improve carbamates diffusion into the central nervous system, while the blood brain barrier penetration in the healthy body is prevented [76].

Some carbamate compounds are used as herbicides such as ferbam, mancozeb, thiram (Fig. **8**). In addition, carbamates exhibit fungicide activity as well – butylate, pebulate, metham, molinate, cycloate, vernolate (Fig. **8**). It is generally thought that their acute toxicity to humans is low, but they may irritate skin, eyes and throat causing sneezing and coughing [67].

Carbamates, due to their reversible AChE inhibitory action, found an important application in human medicine as pharmacologically active compounds. Natural carbamate derivate physostigmine (Fig. **9**), the secondary metabolite in the plant *Physostigma venenosum*, is widely used in the treatment of myasthenia gravis. As a potent AChE inhibitor, this therapeutic agent reduces ACh hydrolysis rate, and thereby increases its level in damaged neurosynaptic clefts improving nerve impulse transmission. Besides, pyridostigmine (Fig. **9**) is capable to prevent the irreversible binding of OP to AChE. Consequently, it is applied as a prophylactic against nerve agent intoxication [77-79]. Furthermore, rivastigmine (Fig. **4**) is a carbamate with probably the most meaningful pharmacological application, being validated in the symptomatic treatment of AD (see above). 

### 2.2 Irreversible Acetylcholinesterase Inhibitors – Organophosphorus Compounds

OPs are esters or thiols derived from phosphoric, phosphonic, phosphinic or phosphoramidic acid (Fig. **10**).

R_1_ and R_2_ are aryl or alkyl groups that are bonded to the phosphorus atom either directly (forming phosphinates), or through an oxygen or sulphur atom (forming phosphates or phosphothioates). In some cases, R_1_ is directly bonded to the phosphorus atom, and R_2_ is bonded to an oxygen or sulphur atom (forming phosphonates or thiophosphonates). In phosphoramidates, at least one of these groups is –NH_2_ (un-, mono- or bi-substituted), and the atom double-bonded with phosphorus is either oxygen or sulphur. The –X group, also binding to the phosphorus atom through oxygen or sulphur atom, may belong to a wide range of halogen, aliphatic, aromatic or heterocyclic groups. This ″leaving group″ is released from the phosphorus atom when the OP is hydrolyzed by phosphotriesterases or upon interaction with protein targets. In medicine and agriculture, the word ″organophosphates″ refers to a group of insecticides and nerve agents that inhibit AChE [52, 71, 80].

**Fig. (6):**
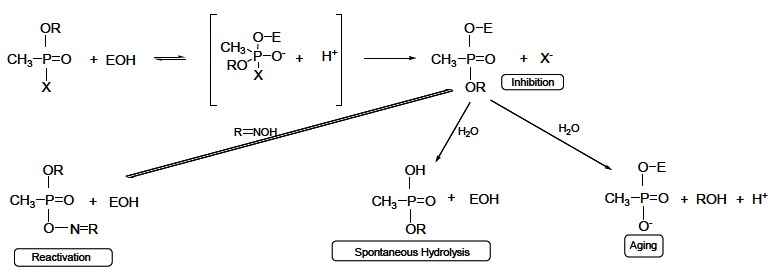
Mechanism of AChE inhibition induced by OPs; reactivation, spontaneous hydrolysis, and aging of the phosphorylated enzyme.

**Fig. (7):**
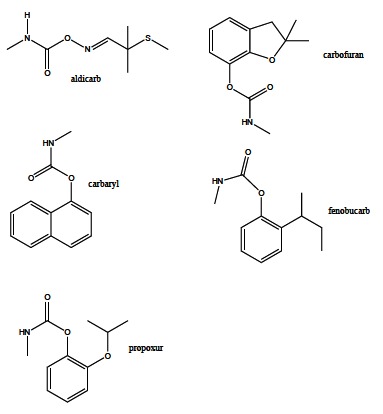
Selected carbamate insecticides.

**Fig. (8):**
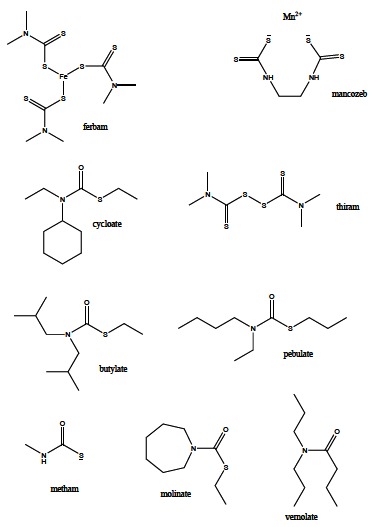
Selected carbamates being applied as herbicides and fungicides.

**Fig. (9):**
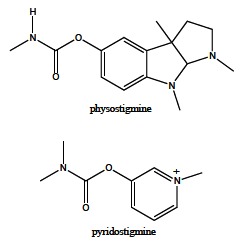
Structural formula of physostigmine and pyridostigmine.

The OPs exert their main toxicological effects through non-reversible phosphorylation of esterases in the central nervous system [81, 82]. The acute toxic effects are related to irreversible inactivation of AChE [82]. Actually, OPs are substrate analogues to ACh, and like natural substrate enter the active site covalently binding to serine –OH group. As in acetylation, OP is split and the enzyme is phosphorylated (Fig. **6**). While the acyl enzyme is quickly hydrolyzed to regenerate the free enzyme, dephosphorylation is very slow (on the order of days), and phosphorylated enzyme cannot hydrolyze the neurotransmitter [83]. The inhibition of the enzyme leads to accumulation of ACh in the synaptic cleft resulting in over-stimulation of nicotinic and muscarinic ACh receptors and impeded neurotransmission. The typical symptoms of acute poisoning are agitation, muscle weakness, muscle fasciculations, miosis, hypersalivation, sweating. Severe poisonings may cause respiratory failure, unconsciousness, confusion, convulsions and/or death [82, 84-86].

Mechanism of OPs induced AChE inhibition is presented using the reaction scheme:

$$E+\mathrm{PX}⇄E^{*}\mathrm{PX}\overset{k_{3}}{\to }\mathrm{EP}+X$$

where, E–enzyme, PX–OP, E^*^PX–reversible enzyme-OP complex, EP–phosphorylated enzyme, X–OP leaving group [87].

Irreversible inhibition occurs in two steps; the first one is fast, short term reversible enzyme inactivation, and its influence is dominant in the begining of the inhibition. The next step is slow irreversible inhibition producing a very stable enzyme-inhibitor complex (phosphorylated enzyme)-inhibitor is covalently bonded to the enzyme [88]. Time dependent irreversible inhibition can be described by the equation:

(1) $$\mathrm{In}\frac{E}{E_{0}}=-\frac{k_{3}t}{1+K_{I}/\left(I\right)}$$

where, E/Eo–remaining enzyme activity related to initial enzyme activity (control) (E_o_), K_I_–dissociation constant for enzyme-inhibitor complex E^*^PX, k_3_–the first rate constant for the conversion of the reversible enzyme-inhibitor complex to phosphorylated enzyme, EP, (I)–inhibitor (OP) concentration, t–time interval after the enzyme and inhibitor mixing. If (I) » (E_o_), the reciprocal slope value of linear dependence ln(E/Eo) - t (Fig. **11a**) can be presented in the form:

(2) $$\frac{1}{k_{\mathrm{app}}}=\frac{1}{k_{3}}+\frac{K_{I}}{k_{3}}\cdot \frac{1}{\left(I\right)}$$

The values of inhibition parameters, K_I_ and k_3_, are calculated from the slope and intersection of 1/k_app_ - 1/(I) linear dependence (Fig. **11b**) [89,90].

**Fig. (10):**
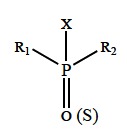
General structural formula of OPs.

**Fig. (11):**
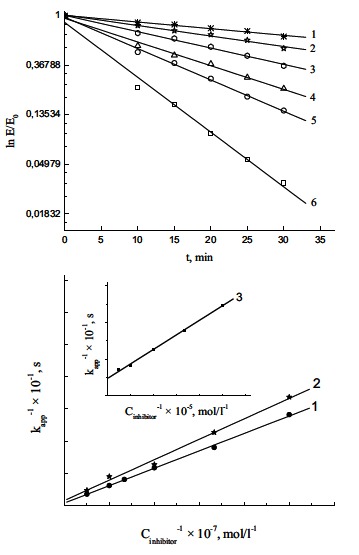
(**a**). Progressive development of inhibition produced by reaction of AChE with different concentrations of diazoxon plotted as semi logarithmic curve in accordance with Equation (1). Diazoxon concentrations (in mol/l): (1) 2 × 10^-8^, (2) 3 × 10^-8^, (3) 5 × 10^-8^, (4) 7.5 × 10^-8^, (5) 1 × 10^-7^, and (6) 2 × 10^-7^. Reproduced from [90]. (**b**). The dependence of kapp upon the concentration of diazoxon (1), chlorpyrifos-oxon (2) and chlorpyrifos ((3), inset) plotted as reciprocals in accordance with Equation (2). Reproduced from [90].

Effective OPs have the following structural features: a terminal oxygen connected to phosphorus by a double bond (oxo form), two lipophilic groups (–R_1_, –R_2_) bonded to the phosphorus, and a good leaving group (–X) bonded to the phosphorus (Fig. **10**) [91].

OPs can produce delayed neurotoxic effect in humans and chickens, called OP induced delayed neuropathy. It is associated with phosphorylation and further dealkylation (aging) (Fig. **6**) of a protein in neurons called neuropathy target esterase, subsequently leading to this syndrome. The symptoms of this neuropathy are paralysis and ataxia, and appear between 14 and 24 days after the poisoning [67, 70, 71]. 

#### 2.2.1 Organophosphorus Insecticides

**Fig. (12):**
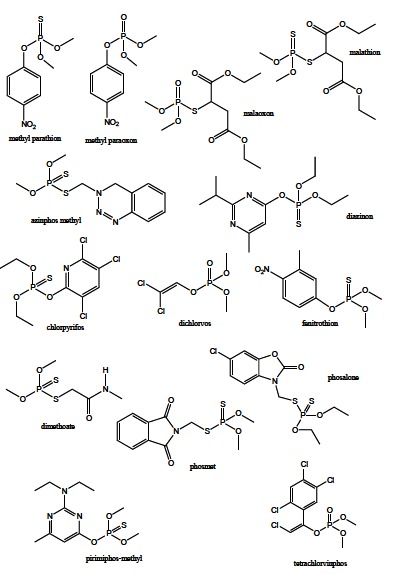
Selected OP insecticides.

The majority of OPs have been commonly used as nonspecific insecticides for over fifty years, to control a variety of insects in agriculture and the household environment. The synthesis of OP pesticides in large quantities started after World War II, and parathion was among the first marketed, followed by malathion and azinphosmethyl. Commonly used OP insecticides have included ethyl parathion, malathion, methyl parathion, chlorpyrifos, diazinon, dichlorvos, phosmet, fenitrothion, tetrachlorvinphos, azinphos methyl, pirimiphos-methyl, dimethoate, phosalone (Fig. **12**) [74, 75, 91-93]. In the 1970s organochlorine insecticides (DDT, dieldrin, aheptachlor) were banned because of their persistence and accumulation in the environment, and replaced by more degradable OPs. 

Actually, OP insecticides in the environment undergo the natural degradation pathway including mainly homogeneous and heterogeneous hydrolysis (especially at high pH) enhanced by the presence of dissolved metals, humic substances, microorganisms and other compounds present in soil [94-96]. OP degradation processes also occur in chemical treatments for purification of polluted waters, generally referred as advanced oxidation processes, as well as throughout the enzymatic reactions in birds, fish, insects and mammals. Degradation studies revealed different kinetics, mechanisms and transformation products, suggesting complete mineralization of the starting compound (usually thio form), but forming toxic break down products as well [89, 97-100]. Actually, oxidation and isomerisation reaction products were reported as much more potent AChE inhibitors compared to the starting thio OPs, while hydrolysis products do not noticeably affect the enzyme activity. Inhibition parameters, IC_50_ and K_i_, for diazinon, malathion, chlorpyrifos and their transformation products are given in Table **1**, indicating even several hundred times lower IC_50_ values for oxo and iso forms related to the thio compounds, and non-inhibiting hydrolysis products [89, 90, 101]. 

Although OPs insecticides degrade rapidly, that made them an attractive alternative to the organochloride pesticides, they have greater acute toxicity, posing risks to people who may be exposed to large amounts - workers employed in the manufacture and application of these pesticides. OPs are one of the most common causes of poisoning worldwide occurring as a result of agricultural use, suicide or accidental exposure. OP pesticides can be absorbed by all routes, including inhalation, ingestion, and dermal absorption [102]. Their toxicity is not limited to the acute phase, but chronic effects have long been noted. Actually, repeated or prolonged exposure to OPs may result in the same effects as acute exposure including the delayed symptoms. The effects, reported in workers repeatedly exposed, include impaired memory and concentration, disorientation, severe depressions, irritability, confusion, headache, speech difficulties, delayed reaction times, nightmares, sleepwalking and drowsiness or insomnia. Influenza-like condition with headache, nausea, weakness, loss of appetite, and malaise has also been reported [103]. Neurotransmitters such as ACh are profoundly important in the brain's development, and many OPs have neurotoxic effects on developing organisms, even from low levels of exposure, causing various diseases of nervous and immune system [85, 104].

Oxo forms of OP insecticides,are highly, approximately equally toxic to warm-blooded as well as cold-blooded organisms. On the other hand, thio forms are converted into the oxo forms by mixed function oxidases. The activation proceeds in cold-blooded organisms but this is not common in warm-blooded organisms where dealkylation into non toxic compounds takes place [51, 105]. Thus, numerous derivatives of highly toxic insecticides have been prepared to reduce the toxicity towards warm-blooded organisms and retain toxicity to insects, thereby enhancing their specificity. The examples of effective, commonly used OP insecticides, and relative safe for warm-blooded organisms are: malathion, chlorpyrifos, fenitrothion, pirimiphos-methyl, dimethoate, phosalone [51].

Nowadays, the common use of OP insecticides results in their accumulation, environmental pollution and acute and chronic poisoning events [106]. For this reason, the use of OP insecticides has to be strictly controlled and restricted. Accordingly, the majority of countries have strong regulations on the application of pesticides; e.g. in the European Union it is regulated by the directive 91/41/EHS [51]. Also, the applied insecticides and their by-products in the environment, water and food are monitored applying different bioanalytical techniques [107]. 

#### 2.2.2 Organophosphorus Nerve Agents/Gases 

**Fig. (13):**
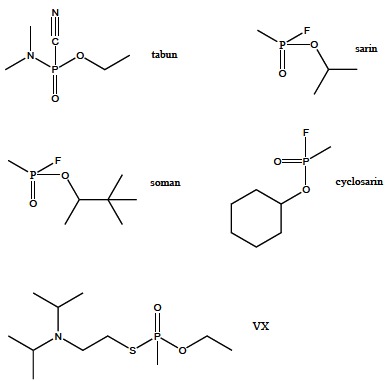
Selected nerve agents.

Nerve agents of OP group include tabun, sarin, soman, cyclosarin and VX. Sarin, soman and cyclosarin are phosphonofluoridates, and VX is a phosphonothioate (Fig. **13**). Soman has four, while sarin and VX have two isoforms, which significantly differ in toxicity and irreversible AChE inactivation rate. Based on the acute toxicity, VX is the most toxic compound among all the nerve agents [67]. The developing and production of these extremely toxic nerve agents started in the 1930s, and later used in wars and by terrorists on several occasions. As chemical weapons, they are classified as weapons of mass destruction by the United Nations, and their production and stockpiling was outlawed by the Chemical Weapons Convention.

Acute poisoning by a nerve agent leads to contraction of pupils, profuse salivation, convulsions, involuntary urination and defecation, and eventual death by asphyxiation as control is lost over respiratory muscles. Some nerve agents are readily vaporized or aerosolized and the primary portal of entry into the body is the respiratory system. Nerve agents can also be absorbed through the skin, requiring that those exposed to such agents wear a full body suit in addition to a respirator [108]. Moreover, the effects of nerve agents are very long lasting and cumulative (increased by successive exposures), and survivors of nerve agent poisoning usually suffer chronic neurological damage that can lead to continuing psychiatric effects [109].

#### 2.2.3 Irreversible Acetylcholinesterase Inhibitors as Therapeutic Agents

OPs, except their use as toxic compounds, have been applied in ophthalmology as therapeutic agents in the treatment of chronic glaucoma, an eye disease in which the optic nerve is damaged in a characteristic pattern. The disease is associated with increased fluid pressure in the eye, and can permanently damage vision in the affected eye(s) and lead to blindness if left untreated [110]. These medical useful OPs include diisopropyl fluorophosphate and echothiophate.

**Fig. (14):**
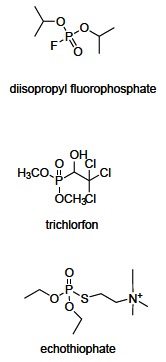
Pharmacologically important OPs.

Diisopropyl fluorophosphate (DFP, DIFP, diisopropyl phosphorofluoridate) (Fig. **14**) is a parasympathomimetic drug, irreversible anti-cholinesterase, and has been used locally in the oily eye drops form as a miotic agent in the glaucoma treatment. It is known as fluostigmine and dyflos in such uses. It exerts ocular side effects mainly associated with its AChE inhibitory properties, and ability to induce delayed peripheral neuropathy [67, 111].

Echothiophate (phospholine) (Fig. **14**) is a parasympathomimetic phosphorothioate, irreversible AChE inhibitor. It is used as an ocular antihypertensive in the treatment of chronic glaucoma and, in some cases, accommodative esotropia. Its application is local (eye drops), and the effects can last a week or more. The drug is available under several trade names such as phospholine iodide. Adverse effects include muscle spasm and other systemic effects [112].

OP compounds may be used in the therapy of neurological damages such as AD and Parkinson's disease. The example is trichlorfon (metrifonate) (Fig. **14**) that used to be applied as a pesticide, and has medicine implementation analogous to the carbamate rivastigmine (described above) [113].

The described reversible and irreversible AChE inhibitors are summarized in Table **2**.

#### 2.2.4. Nonspecific Toxic Effects of Organophosphates

The primary target of OP action is AChE, and the main mechanism of toxicity in acute OP exposure involves the specific irreversible inhibition of this enzyme activity in the nervous system and blood, manifesting as a cholinergic crisis with excessive glandular secretions and weakness, miosis and fasciculation of muscle, which may lead to death [106, 114, 115]. Additionally, many studies suggest that both acute and chronic intoxication disturb the redox processes changing the activities of antioxidative enzymes and causing enhancement of lipid peroxidation in many organs, and there is little correlation between organ damage and the degree of OP induced AChE inhibition [116-118]. Indeed, in acute, and rather subchronic or chronic OP exposition, induction of oxidative stress has been reported as the main mechanism of its toxicity [119]. Oxidative stress is defined as an imbalance between the production of free radicals – reactive oxygen species (ROS) and the antioxidant defense system – enzymatic and non-enzymatic. The ROS may be generated as the result of the metabolism of OPs by cytochrome P450s, monooxygenases that catalyze oxidation by addition of one atom of molecular oxygen into the substrate (OP) by electron transport pathway [120]. This disorder is manifested by changes in the activity of antioxidative enzymes (catalase, superoxide dismutase, glutathione peroxidase, glutathione reductase), increased malondialdehyde concentration and/or altered levels of non-enzymatic antioxidants (reduced glutathione, vitamin C, vitamin E, beta-carotene), as oxidative stress parameters and the marker of ROS increased level and lipid peroxidation [99, 121, 122]. There is some evidence that OPs may affect liver, kidney, muscles, immune, and hematological system, causing many human body disorders [123-125]. Also, some findings indicate oxidative stress as an important pathomechanism of neurological disorders such as AD and Parkinson's disease, as well as of cardiovascular diseases [122, 126, 127]. 

Furthermore, OP induced ROS attack lipids, proteins and DNA, causing oxidation and membrane damage, enzyme inactivation, DNA damage and cell death [128-130]. The highly reactive free radicals attack DNA resulting in single and double strand breaks, as well as oxidative damage to sugar and base residues that can later be converted to strand breaks [131]. On the other hand, phosphorus moiety in the OPs appears to be a good substrate for nucleophilic attack leading to phosphorylation of DNA which is an instance of DNA damage [132]. Some reported studies indicate the increase in chromosomal aberrations (CA), micronuclei (MN) and sister chromatid exchanges (SCE), as the markers of cytogenetic damage, in cultured lymphocytes isolated from peripheral blood taken from exposed individuals. Thus, cytogenetic damage in circulating lymphocytes has been widely used as a biomarker of exposure and effects of pesticides [133, 134]. It has been reported that AChE non-inhibiting OP decomposition products exert stronger genotoxic potency compared to the parent compound [99], suggesting that the risk of genotoxicity from some insecticides might be appreciably greater than that predicted from standard toxicity tests [135]. Moreover, DNA damage leads to genomic instability that may result in mutagenesis and carcinogenesis [136]. Some epidemiological studies demonstrate cancer risk due to pesticides exposure [137-139], while The United States Environmental Protection Agency lists parathion as a possible human carcinogen [140].

## 3 DETOXIFICATION OF ACETYLCHOLINESTERASE INHIBITORS

### 3.1 Pharmacological Treatment of Organophosphates Induced Intoxication

**Fig. (15):**
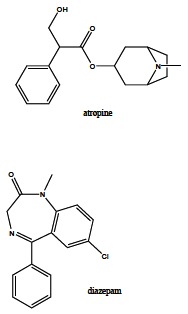
Frequently used drugs for symptomatic treatment of OP intoxication.

**Fig. (16):**
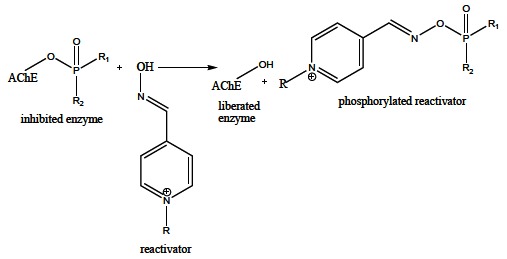
Regeneration of inhibited AChE activity by oxime reactivators.

The mechanism of OP insecticides action is based on the irreversible inhibition of AChE in an insect body, resulting in the disrupted neuronal transmission and the consequent death. However, the OPs are not selective for insect species, but they have the same mechanism of action for the warm-blooded organisms including humans that may be also intoxicated. Actually, OPs irreversibly inhibit human AChE in Ser203 leading to the cholinergic crisis which manifests as the muscarinic (lacrimation, salivation, miosis), nicotinic (neuromuscular blockade) or central (breath depression) symptoms, and the organism death in the case of untreated OP intoxication [52]. OP poisoning can be treated non-pharmacologically and pharmacologically [141]. The non-pharmacologic treatment includes resuscitation, oxygen supply or decontamination depending on the OP entrance to the human body (*e.g.* skin, eye, gastric decontamination) [142], whereas the pharmacologic treatment is based on the administration of symptomatic and causal drugs. Parasympatolytics (usually atropine, Fig. **15**), that reduce the effects of the accumulated ACh on the cholinergic receptors [143], and anticonvulsives (usually diazepam, Fig. **15**), diminishing neuromuscular seizures [144], are used as the symptomatic treatment. The causal treatment comprises AChE reactivators that, unlike the symptomatic drugs, regenerate the enzyme native function by cleaving OP moiety from AChE serine active site. The mechanism of AChE reactivation is based on the nucleophilic attack of the reactivator hydroxyiminomethyl (oxime) moiety towards the OP moiety of the phosphorylated AChE. The covalent bond between OP and AChE serine is cleaved, reactivator-OP complex (phosphorylated reactivator) is formed, and the enzyme is liberated (Fig. **16**) [145, 146]. However, the inactivated phosphorylated enzyme can be dealkylated (Fig. **6**), and such aged OP-AChE complex cannot be reactivated by the oxime reactivators. Therefore, the oxime reactivators should be administered rapidly after the OP poisoning [147].

**Fig. (17):**
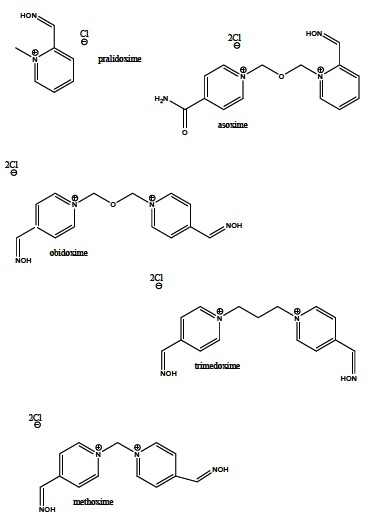
Commercially available oxime reactivators of AChE activity.

The commercially available oxime reactivators - pralidoxime, methoxime, trimedoxime, obidoxime, asoxime (Fig. **17**) were developed in 1950s, and primarily intended to reduce the nerve agents intoxication [148]. Additionally, some of them exerted good effects in the restoration of inhibited cholinesterases by OP insecticides as well [149]. Although pralidoxime was the first synthesized and most common used causal drug, its capability to regenerate the inhibited enzyme activity is not satisfactory. On the contrary, bisquaternary oximes, trimedoxime and obidoxime exhibited very good abilities in the treatment of OP insecticides poisoning, whereas asoxime was found to be effective for nerve agent induced intoxication and without the meaningful reactivation of the inhibited AChE by OP insecticides. Moreover, obidoxime, combined with atropine and diazepam, has demonstrated positive results in the clinical trials [150, 151]. Furthermore, among 300 synthesized and evaluated oxime reactivators, some novel compounds (*e.g.* K027, BT-07-4M, TMB-4, BT-08) possess, related to the commercially available reactivators, both better reactivation capabilities against different OP insecticides and significantly decreased toxicity tested using *in vitro *and *in vivo *animal models [152-154]. 

### 3.2 Detoxification of Organophosphorus and Carbamate Insecticides Through Enzymatic Hydrolysis

Carbamates and OPs are apolar compounds accumulating in fatty tissues, and can be eliminated by their conversion to water soluble compounds. The most effective way to increase the water solubility of these toxic compounds is hydrolysis to much more water soluble metabolites that may be removed in urine. Although these insecticides are able to hydrolyze spontaneously especially at high pH, the main route of their detoxification is enzymatic degradation by hydrolases generating less toxic metabolites. 

**Fig. (18):**
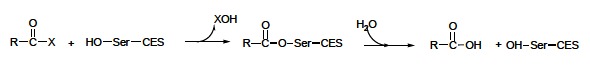
Mechanism of CESs catalyzed hydrolysis of carboxyl esters.

Carbamates are most effectively decomposed by carboxylesterases (CESs), the esterases capable to hydrolyze carboxyl esters [155]. The mechanism of the CESs catalyzed hydrolysis of carboxyl esters (Fig. **18**) consists in the reversible acylation of a serine residue in the active centre of the protein, releasing the covalently acylated enzyme and the alcohol moiety of the carboxyl ester. Subsequently, the acylated intermediate is decomposed by nucleophilic water attack to the corresponding carboxylic acid and the free active CES participating in the new catalytic cycle. Highest CESs concentrations have been found in serum and liver of mammals [156].

**Fig. (19):**
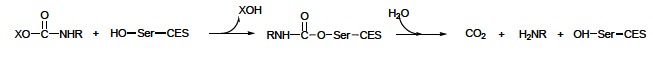
CESs catalyzed hydrolysis of carbamate insecticides.

Since the carbamates are structurally similar to carboxyl esters, these insecticides are susceptive to CESs catalyzed hydrolysis. The mechanism of carbamates induced inhibition of CESs is similar to the mechanism of hydrolysis of the enzyme natural substrates. The additional step is the final rapid degradation of carbamic acid to carbon dioxide and the corresponding amine (Fig. **19**). 

Moreover, oxidized metabolites of the parent carbamate insecticides can also be decomposed by CESs due to the intact carbamic ester bond [71]. The metabolism of carbamates depends on their chemical structure and animal species. So, the hydrolysis of carbaryl in rabbit serum is significantly more efficient than chicken and human serum activity. Also, CESs with the ability to hydrolyze carbamates have been found in some bacteria (*Blastobacter, Arthrobacter, Pseudomonas,* *Achromobacter,* *Micrococcus)* [71, 157]. Additionally, some findings have demonstrated the capacity of serum albumin to decompose *N*-methylcarbamates and some OPs, and its hydrolyzing potency differs among species. Generally, mammals exert higher hydrolyzing activity than birds [71, 158, 159]. 

**Fig. (20):**
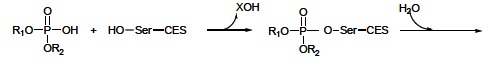
Inhibition of B-esterases by OPs.

OPs are detoxified through oxidation and hydrolysis. The enzymes included in the hydrolysis of these toxic insecticides are CESs and phosphotriesterases (PTEs). Actually, in mammals (especially in liver and serum) there is a pool of CESs of unknown physiological role, named B-esterases that both hydrolyze carboxyl esters and being inhibited by OPs. Different from A-esterases being capable to hydrolyze carboxyl esters but not inhibited by OPs, B-esterases may be involved in the detoxification of OPs, and carbamates as well [160]. While OP and carbamate insecticides exhibit major toxic effects through phosphorylation and carbamylation, respectively, of AChE and neuropathy target esterase inactivating these CESs, the inhibition of B-esterases is not associated with evident toxic effects. The mechanism of B-esterases inhibition by OPs is analogous to that induced by carbamates (Fig. **19**). The difference is in the final step; the phosphorylated enzyme cannot be reactivated by water, and cannot release the free active enzyme (Fig. **20**). In this detoxification system that removes the insecticides from the media, each molecule of CESs scavenges at least one molecule of the toxic compound before this reaches targets in the nervous system [71, 161]. 

**Fig. (21):**
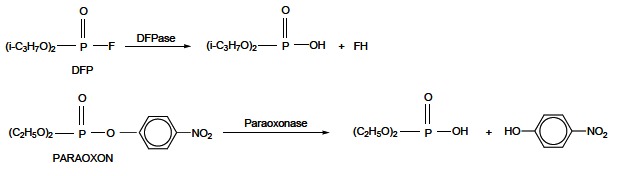
OPs hydrolysis catalyzed by known PTEs: DFPase and paraoxonase.

However, much more efficient route of OPs detoxification is hydrolysis by PTEs, esterases of an unknown physiological role. The mechanism of PTEs action is based on the bond cleavage between the phosphorous atom and the leaving group (–X) of OPs (Fig. **21**). The resulting metabolites are less toxic and more polar than the parent OP, and therefore do not accumulate in fatty tissues and are eliminated in urine. Unlike OPs detoxification by CESs where one molecule of CESs scavenges and removes one molecule of OP, one molecule of PTE is able to degrade plenty of OP molecules, which makes this detoxification route more sovereign [162]. PTEs have been found in various biological tissues of mammals, fish, birds, molluscs and bacteria. Although these detoxification enzymes are located in a few tissues of mammals, higher PTEs activity levels have been detected in serum and liver [158, 163]. 

Some studies indicate correlation between PTEs presence in some species and their susceptibility to toxic effects of OPs. So, insects and birds lack paraoxonase, whereas mammals contain high concentrations of this PTE making them more resistant to paraoxone toxicity [163]. For the strong potency to degrade the toxic insecticides, exogenous purified PTEs exert protective effects against OPs poisoning and can be used as prophylactics and antidotes in the therapeutic treatment of OPs intoxication [164-166]. Besides, these decomposing enzymes exhibit promising applications in other biotechnology fields: bioremediation of wasted materials, biodegradation of insecticide residues, detoxifying warfare arsenals, development of biosensors for OPs [167, 168].

## 4 FINAL REMARKS

The pharmacological and toxic actions of AChE inhibitors consist in inactivation of the enzyme activity resulting in accumulation of synaptic ACh and consequent enhanced stimulation of postsynaptic cholinergic receptors in the central and/or peripheral nervous systems. According to the mode of action, AChE inhibitors can be divided into two groups: irreversible and reversible**. **Reversible, competitive or noncompetitive, inhibitors (donepezil, rivastigmine, galantamine) are protagonists in the pharmacotherapy of AD symptoms. Their therapeutic effect is based on maintaining ACh level through slowing down its hydrolysis rate. Hence, these generally well tolerated drugs enhance cholinergic neurotransmission in forebrain regions and compensate for the loss of functioning brain cells. In addition to AD, reversible AChE inhibitors of various chemical structures have noteworthy pharmacological application in the treatment of neurological disorders manifestations such as myasthenia gravis, Lewy bodies and Parkinson’s disease dementia, and as prophylactics against nerve agent intoxication as well. On the other hand, carbamate reversible ACh inhibitors also exhibit toxic mode of action, and are used as insecticides, fungicides and herbicides. Different from reversible inhibitors, irreversible AChE inactivators including OPs have toxicological relevance accompanied with ACh accumulation in the synaptic cleft and disrupted neurotransmission. Accordingly, these toxic compounds are applied as nerve gases, and insecticides being most frequently used in last two decades. OPs, as well as carbamate pesticides, can be detoxified through enzymatic hydrolysis in mammals. Carbamates, as structural analogs to carboxyl esters, mainly undergo CESs catalyzed degradation, whereas the most efficient detoxification route of OPs is the hydrolysis catalyzed by different PTEs. Besides, OPs induced poisoning can be pharmacologically treated by simptomatic (atropine, diazepam), and causal drugs such as oxime reactivators. Except their use as toxic compounds, some OPs demonstrate pharmacological significance. For instance, diisopropyl fluorophosphate and echothiophate have been administered in the treatment of chronic glaucoma. Although the use of AChE inhibitors relies on the interaction with AChE as their primary target, a number of cholinesterase inhibitors such as OPs have additional sites of action that may have toxicological relevance. Actually, acute, and rather subchronic or chronic OP exposition results in elevated ROS level attacking lipids, proteins, DNA, and consequently causes membrane damage, enzyme inactivation, DNA damage and cell death.

## Figures and Tables

**Table 1:** IC_50_ and K_I_ Values for Irreversible Inhibition of AChE Activity by Diazinon, Chlorpyrifos, Malathion, and their Transformation Products

Compound	IC_50_ (20 min), mol/L [75]	K_i_, mol/L [75]	Compound	IC_50 _(5 min), mol/L [86]	K_i_, mol/L [74]
Diazinon	> 2.0 × 10^-4^	/ **	Malathion	3.2 × 10^-5^	1.3 × 10^-4^
Diazoxon	5.1 × 10^-8^	7.9 × 10^-7^	Malaoxon	4.7 × 10^-7^	5.6 × 10^-6^
IMP*	/ ***	/ ***	Isomalathion	6.0 × 10^-7^	7.2 × 10^-6^
Chlorpyrifos	4.3 × 10^-6^	9.6 × 10^-6^	Diethylmaleate	6.0 × 10^-2^	/ ***
Chlorpyrifos-oxon	3.0 × 10^-8^	4.3 × 10^-7^	O,O-dimethyl thiophosphate	/ ***	/ ***
3,5,6,-trichloro-2-pyridinol	/ ***	/ ***			

* 2-isopropyl-6-methyl-4-pyrimidinol

** the data is unavailable

*** compound does not inhibit AchE

**Table 2:** Commonly used Reversible and Irreversible AChE Inhibitors and their Application

Compound	Chemical Structure	Mode of AChE Inhibition	Application

Donepezil	Piperidine derivative	Reversible	Alzheimer's disease
			Autism

Rivastigmine	Carbamate	Reversible	Alzheimer's disease
			Lewy bodies
			Parkinson's disease

Galantamine	Alkaloid	Reversible	Alzheimer's disease

Tacrine	Pyridine derivative	Reversible	Alzheimer's disease

7-methoxytacrine	Pyridine derivative	Reversible	Alzheimer's disease

Huperzine A	Alkaloid	Reversible	Alzheimer's disease

Aldicarb	Carbamate	Reversible	Insecticide
Carbofuran			
Carbofuran			
Carbaryl			
Propoxur			

Ferbam	Carbamate	Reversible	Herbicide
Mancozeb			
Thiram			

Butylate	Carbamate	Reversible	Fungicide
Pebulate			
Metham			
Molinate			
Cycloate			
Vernolate			

Physostigmine	Carbamate	Reversible	Myasthenia gravis

Pyridostigmine	Carbamate	Reversible	Prophylactic against nerve agent intoxication

Ethyl parathion		Irreversible	Insecticide
Malathion	Organophosphorus compound		
Methyl parathion			
Chlorpyrifos			
Diazinon			
Dichlorvos			
Phosmet			
Fenitrothion			
Tetrachlorvinphos			
Azinphos methyl			
Pirimiphos methyl			
Dimethoate			
Phosalone			

Tabun	Organophosphorus compound	Irreversible	Nerve agent
Sarin			
Soman			
Cyclosarin			
VX			

Diisopropyl Fluorophosphate	Organophosphorus compound	Irreversible	Glaucoma

Diisopropyl Fluorophosphate	Organophosphorus compound	Irreversible	Glaucoma

Echothiophate	Organophosphorus compound	Irreversible	Glaucoma
			Accommodative esotropia

Trichlorfon	Organophosphorus compound	Irreversible	Alzheimer's disease
			Parkinson’s disease

## ABBREVIATIONS

ACh: = acetylcholine
AChE: = acetylcholinesterase
AD: = Alzheimer's disease
BuChE: = pseudocholinesterase; butyrylcholinesterase
CA: = chromosomal aberrations
CES(s): = carboxylesterase(s)
DNA: = deoxyribonucleic acid
EMA: = European Medicines Agency
FDA: = U.S. Food and Drug Administration
IMP: = 2-isopropyl-6-methyl-4-pyrimidinol
MN: = micronuclei
NMDA: = N-methyl-D-aspartic acid
OP(s): = organophosphorus compound(s); organophosphate( s)
PTE(s): = phosphotriesterase(s)
ROS: = reactive oxygen species
SCE: = sister chromatid exchanges
